# Analysis of global Napier grass (*Cenchrus purpureus*) collections reveals high genetic diversity among genotypes with some redundancy between collections

**DOI:** 10.1038/s41598-023-41583-7

**Published:** 2023-09-04

**Authors:** Meki S. Muktar, Tadelech Bizuneh, William Anderson, Yilikal Assefa, Alemayehu T. Negawo, Abel Teshome, Ermias Habte, Alice Muchugi, Tileye Feyissa, Chris S. Jones

**Affiliations:** 1 Feed and Forage Development, International Livestock Research Institute, Addis Ababa, Ethiopia; 2 Ethiopian Institute of Agricultural Research, Holeta Agricultural Research Centre, Holeta, Ethiopia; 3 Crop Genetics and Breeding Research Unit, USDA-ARS, 115 Coastal Ways, Tifton, GA 31793 USA; 4 Institute of Biotechnology, College of Natural Sciences, Addis Ababa University, Addis Ababa, Ethiopia; 5 Feed and Forage Development, International Livestock Research Institute, Nairobi, Kenya

**Keywords:** Computational biology and bioinformatics, Genetics, Molecular biology, Plant sciences

## Abstract

Genetic diversity amongst genotypes of several Napier grass collections was analyzed and compared with the diversity in a set of open pollinated progeny plants. A total of 114,881 SNP and 46,293 SilicoDArT genome-wide markers were generated on 574 Napier grass genotypes. Of these, 86% of the SNP and 66% of the SilicoDArT markers were mapped onto the fourteen chromosomes of the Napier grass genome. For genetic diversity analysis, a subset of highly polymorphic and informative SNP markers was filtered using genomic position information, a maximum of 10% missing values, a minimum minor allele frequency of 5%, and a maximum linkage-disequilibrium value of 0.5. Extensive genetic variation, with an average Nei’s genetic distance value of 0.23, was identified in the material. The genotypes clustered into three major and eleven sub-clusters with high levels of genetic variation contained both within (54%) and between (46%) clusters. However, we found that there was low to moderate genetic differentiation among the collections and that some overlap and redundancy occurred between collections. The progeny plants were genetically diverse and divergent from the germplasm collections, with an average *F*_*ST*_ value of 0.08. We also reported QTL regions associated with forage biomass yield based on field phenotype data measured on a subset of the Napier grass collections. The findings of this study offer useful information for Napier grass breeding strategies, enhancement of genetic diversity, and provide a guide for the management and conservation of the collections.

## Introduction

Napier grass (*Cenchrus purpureus* (Schumach.) Morrone syn. *Pennisetum purpureum* Schumach.), also known as elephant grass, is a perennial C4 grass belonging to the genus *Cenchrus*, one of the most diverse genera, consisting of about 140 species including important cultivated species such as Napier grass, pearl millet and kikuyu grass^1–3^. Napier grass is native to Sub-Saharan Africa and has been distributed to other tropical and subtropical regions around the world, adapting to a wide range of agro-ecologies, from sea level to 2500 m above sea level (m.a.s.l.). It has been acclimatized in areas of Central and South America, tropical parts of Asia, Australia, the Middle East and the Pacific islands^1, 4^.

Napier grass is mainly cultivated as a forage crop for animal feed due to its high palatability and feed quality, high dry matter production (up to 78 tons of dry matter/ha/year), and year-round availability under supplementary irrigation^1, 5–7^. In addition, Napier grass has the potential to produce biofuels such as alcohol, ethanol and butanol, and methane since it has high cellulose content that can be used as a carbon (energy) source^8–11^. It has many other beneficial characteristics, including resistance to most pests and diseases, ease of establishment and fast regrowth capacity, withstanding repeated cuttings. It is also resistant to high temperatures, and has low water and nutrient requirements^4, 12^. However, its productivity is primarily checked by the unpredictable weather arising from changes in climatic conditions^13, 14^ and biotic stressors, such as the diseases head smut and Napier stunt, particularly in Central and Eastern African countries^15–17^.

The availability of genetic diversity is a prerequisite for breeding and genetic improvement programs. It serves to identify novel alleles for agronomically important traits, such as disease and pest resistance, drought tolerance, and withstanding challenges associated with the changing climate^1, 18^. Napier grass is a cross-pollinating allotetraploid species with a chromosome number of 2n = 4x = 28 (genome A’A’BB)^19, 20^. The Napier grass A’A’ genome has been reported to be homologous to the AA genome of pearl millet (*Cenchrus americanus* Morrone syn. *Pennisetum glaucum*; 2n = 2x = 14; AA)^21^. The two species are cross-compatible, producing triploid (AA’B genome) sterile hybrids that can only be propagated by vegetative means^1, 22, 23^. The hybrids are favored as a forage as they combine the superior forage quality of pearl millet with the high yielding ability of Napier grass^1^. Consequently, these interspecific triploid hybrids have become a crucial part of the improved forage crop value chain in Africa, Asia and South America^1, 4^.

Several national and international genebanks have conserved a substantial number of Napier grass genotypes collected from various geographical regions. For example, the International Livestock Research Institute (ILRI); the Brazilian Agricultural Research Corporation, Empresa Brasileira de Pesquisa Agropecuária (EMBRAPA), United States Department of Agriculture (USDA-ARS), International Crops Research Institute for the Semi-Arid Tropics (ICRISAT) and the Millennium Seed Bank Kew (https://www.genesys-pgr.org) have collections containing a considerable amount of germplasm. Although Napier grass exhibits high genetic variation due to its strict out crossing and self-incompatibility nature, the global diversity is low due to its vegetative propagation and the common use of cuttings as planting material. This would likely have limited the exchange of genes between genotypes. However, limited information exists about the overall available genetic diversity and germplasm exchange among the global collections.

The self-incompatibility and outcrossing nature of Napier grass^20, 26^ results in a mixed lot of seeds from a genotype, thus progeny plants are heterogeneous and unpredictable in terms of performance^1^. In addition, reports of poor seed-setting, seed shattering, low seed germination, and weak seedlings indicate that seed propagation of this grass would be difficult^4^. However, in our experience the majority of the ILRI Napier grass genotypes growing in the Bishoftu and Ziway conservation sites can produce viable seeds^27^, and their progeny plants have been grown with varying degrees of performance, indicating their potential for breeding and improvement of the crop. Progeny selection from open pollination and targeted crossing have been the approach to breeding Napier grass and have resulted in the production of many improved cultivars^1^. The high biomass producing tall cultivar ‘Merkeron’ and the nutritious dwarf leafy cultivar ‘Mott’, which is more suitable for grazing, are among the well-known cultivars developed from targeted crossing^1, 28–30^.

Wider genetic diversity is an essential source of novel and useful alleles for improvement^31^ and will have an immediate practical impact on genetic improvement for productivity and performance^18^. In this study, we analyzed and compared the genetic diversity between and within Napier grass worldwide collections and progeny plants raised from seeds using high density genome-wide markers. The aim was to assess the overall genetic diversity in the global collections and to compare it with the diversity in progeny plants to generate information useful for designing breeding strategies in Napier grass.

## Results

### Napier grass populations used in the study

With the aim of enhancing the genetic diversity and increasing the population size of the Napier grass collection held in the ILRI forage genebank, we analysed 32 new genotypes from ICRISAT, 93 from the Kenya Agricultural and Livestock Research Organization (KALRO), 86 from EMBRAPA, and 23 from the USDA-ARS collections. The genotypes from the USDA-ARS were selected, based on a previous genetic diversity study on the collection^28^, to represent as much of the diversity as possible held in the USDA-ARS’s collection. In addition, 219 progeny plants were raised from 13 seed setting Napier grass genotypes of the ILRI collection (7 to 22 progeny plants per seed parent) (Supplementary Table S2), with the aim of releasing the genetic diversity held in the collection by dissecting the previously observed long haplotype blocks^18^. An additional 21 genotypes, composed of two commercial varieties, Maralfalfa and Super-Napier (*Cenchrus purpureus* × *Cenchrus americanus*), and other genotypes with unknown or different names, were included. We also included the 60 ILRI and 47 EMBRAPA genotypes, which have been studied previously^18, 32^ to assess and visualize the overall diversity and identify new and unique genotypes. Out of these initially selected 581 Napier grass genotypes, genotypes with high missing values for the genome wide SNP markers were removed and a final set of 574 (Supplementary Table S1) genotypes was studied.

### High density genome-wide DArTseq markers and their polymorphism

Genotyping generated 114,881 SNP and 46,293 SilicoDArT markers, distributed over the Napier grass genome. Approximately 86% (98,535) of the SNP and 66% (30,745) of the SilicoDArT markers were aligned onto the fourteen chromosomes of the Napier grass genome and about 2% (2653 SNPs and 918 SilicoDArTs) of the markers were mapped onto different contigs^19^. The highest number of markers mapped onto chromosome CpA01 (8057 SNPs and 2947 SilicoDArTs) while the lowest number mapped onto CpB07 (4411 SNPs and 1245 SilicoDArTs).

**Figure 1:**
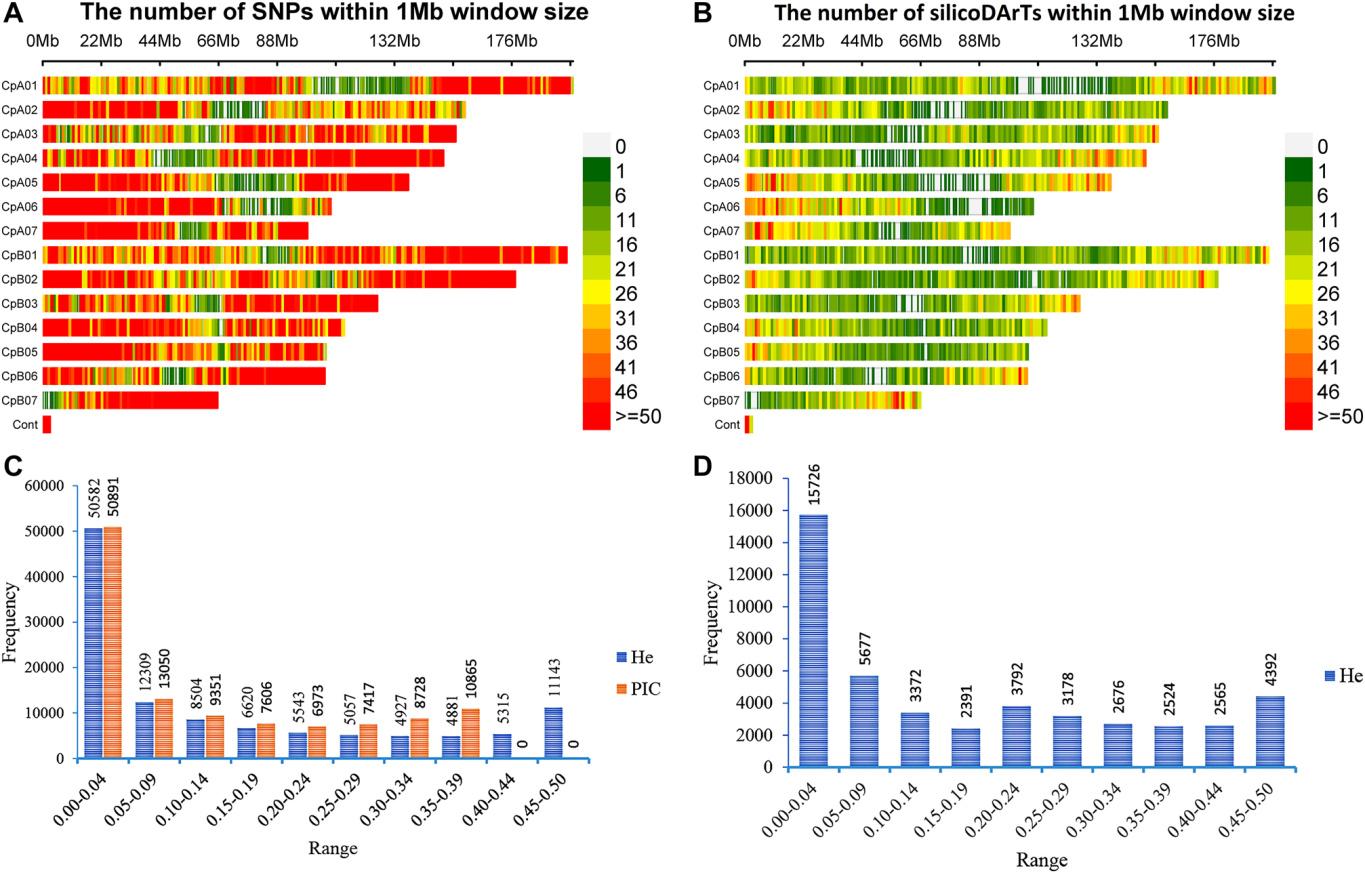
Genome-wide distribution and polymorphism of the markers. The distribution of the markers across the fourteen chromosomes of the Napier grass genome is shown in (**A**) (SNPs) and (**B**) (SilicoDArTs). The distribution of polymorphic information content (PIC) (orange) and heterozygosity (He) (blue) values are shown for the SNPs (**C**) and SilicoDArTs (**D**).

The percentages of the markers missing values ranged from 0 to 22% for the SilicoDArT markers and 0 to 62% for the SNP markers. Heterozygosity (He) values for the SNP markers ranged from 0 to 0.5 with an average value of 0.15 while polymorphic information content (PIC) values ranged from 0 to 0.38 with an average value of 0.13 (Fig. 1). For the SilicoDArT markers, in which there is no difference between PIC and He, the values ranged from 0 to 0.38 with an average value of 0.15. More than 37% of the SilicoDArT markers and 30% of the SNP markers had PIC values above 0.25.

**Figure 2:**
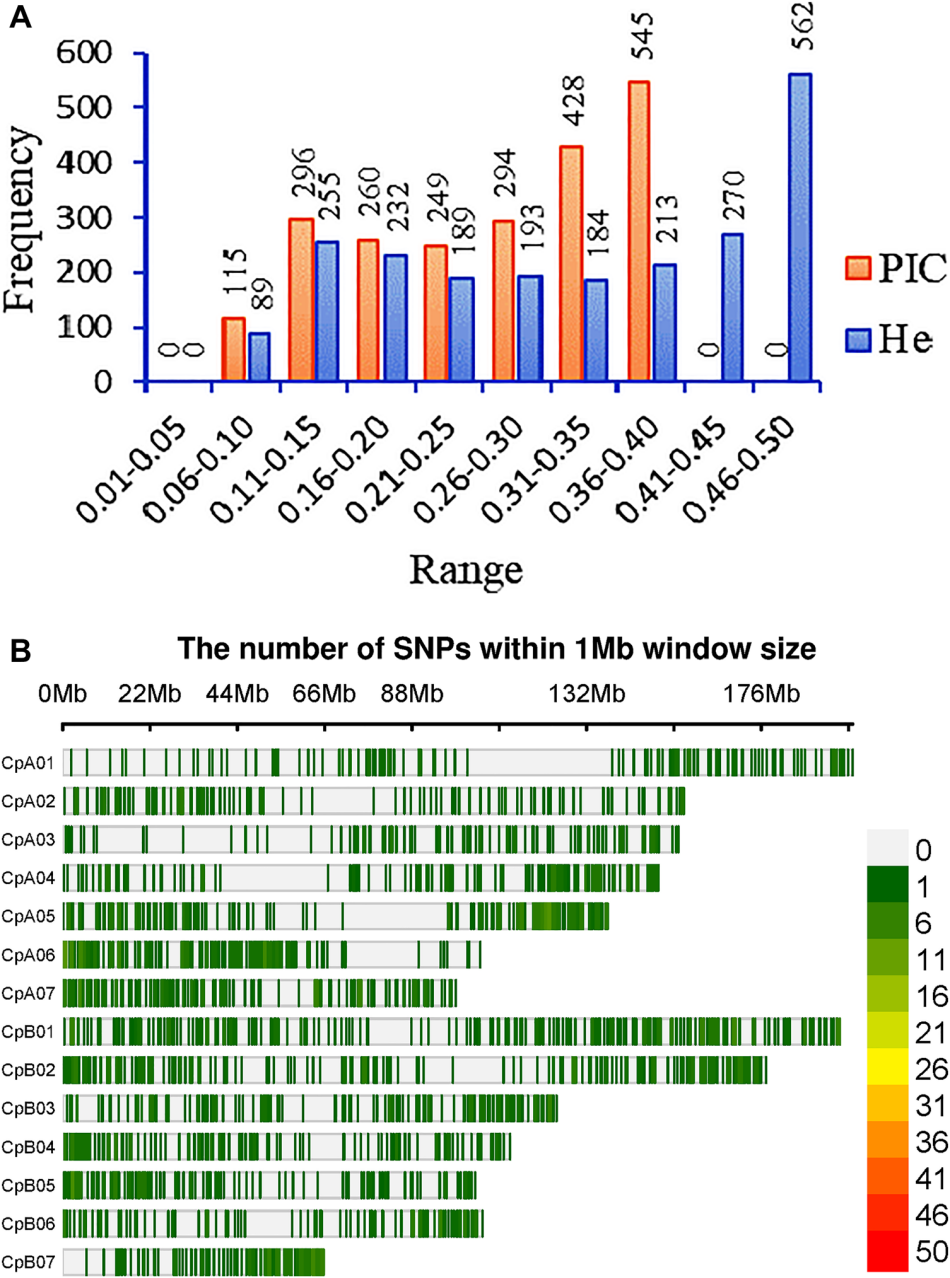
Polymorphism of SNP markers filtered for genetic diversity analysis. The distribution of polymorphic information content (PIC) and heterozygosity (He) of the markers are shown by orange and blue colors, respectively (**A**). Their distribution across the fourteen Napier grass chromosomes is shown in (**B**).

In order to assess the genetic diversity of the collections, a subset of 2,186 robust SNP markers were selected based on the marker’s minor allele frequency (MAF ≥ 5%), missing values (≤ 10%), independence from each other (linkage disequilibrium-LD ≤ 0.5), and their distribution across the Napier grass genome. The PIC and He values of the selected markers ranged from 0.08 to 0.38 and 0.09 to 0.50 with an average value of 0.26 and 0.32, respectively, and about 60% of the markers had PIC and He values above 0.25 (Fig. 2). All the selected markers were able to be mapped onto the Napier grass genome^19^.

### The overall genetic diversity among Napier grass genotypes from different collections

**Figure 3:**
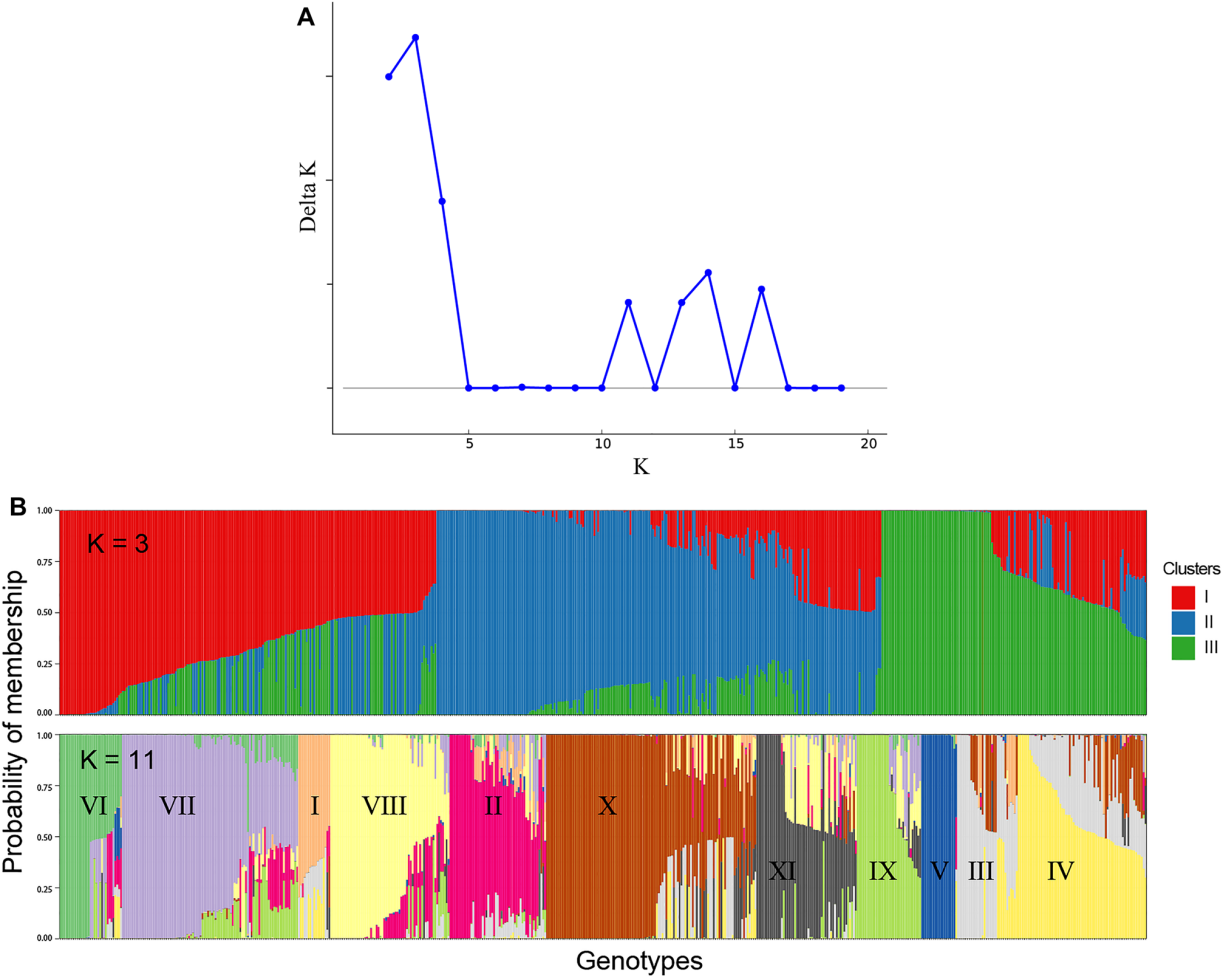

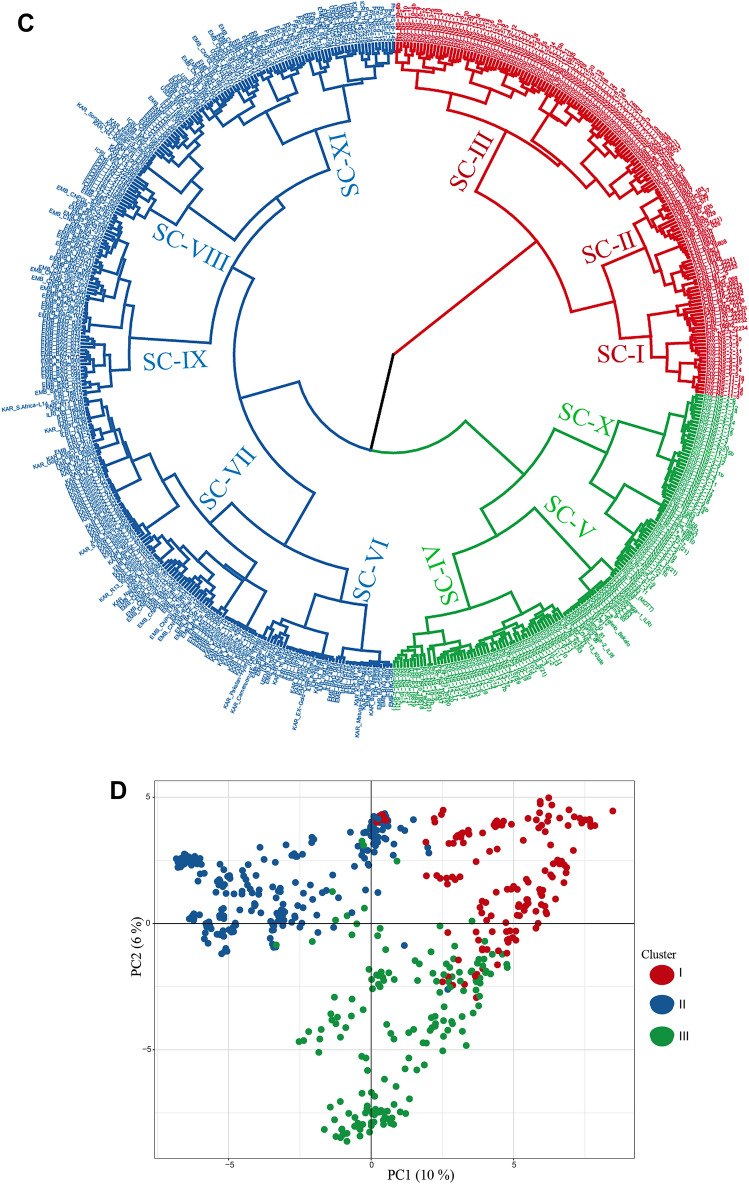
Major clusters and sub-clusters of the Napier grass collections and progeny plants used in the study. (**A**) The delta K suggesting three major clusters that are partitioned into sub-clusters. (**B**) Bar plots based on the admixture model in STRUCTURE, for K = 3 and 11. (**C**) Clusters detected by hierarchical clustering. (**D**) Clusters detected by PCA.

Genetic diversity and population structure in all genotypes from the different collections were analysed using the Bayesian algorithm implemented in STRUCTURE software, hierarchical clustering, and PCA analyses. In the STRUCTURE analysis, the delta K showed the presence of three major and up to sixteen sub-clusters (Fig. 3A,B). At a 60% membership probability threshold of k = 3, 158 genotypes were assigned to Cluster I, 246 genotypes to Cluster II, and 170 genotypes to Cluster III (Supplementary Table S3). Cluster I was composed mainly of genotypes from the ILRI collection (25 out of 59, 42%), progeny plants (83 out of 219, 38%) and the ICRISAT collection (10 out of 26, 38%). In addition, about 20% of genotypes from KALRO (19 out of 93), 11% (15 out of 133) from EMBRAPA, and 9% (2 out of 23) from the USDA-ARS collections were clustered in this group. Cluster II contains the majority of the EMBRAPA (106 out of 133, 86%), KALRO (74 out of 93, 80%), and ILRI (31 out of 59, 53%) collections and about half of the genotypes from the ICRISAT and USDA-ARS collections. Only three progeny plants from NS-12 (Napier grass seedling number 12) were clustered in this group. Cluster III was mostly represented by progeny plants (133 out of 219, 61%) followed by USDA-ARS genotypes (10 out of 23, 43%) and three genotypes from ICRISAT, twelve from EMBRAPA, and three from the ILRI collection. Each major cluster was further partitioned into two to five sub-clusters that made an optimum number of eleven sub-clusters and three admixture groups (Supplementary Table S3). A total of 242 genotypes, composed of 69% of the ICRISAT collection, 49% of the progeny, 47% of the EMBRAPA, 43% of the USDA-ARS, 37% of the ILRI, and 15% of the KALRO collections were under three different admixture groups. The three major clusters and the eleven sub-clusters were also detected by hierarchical clustering, in which the genotypes assignment in each major and the sub-clusters were mostly consistent with the clusters detected by the STRUCTURE analysis (Fig. 3C). A similar result was observed by the PCA, in which the first and second principal components explained about 16% of the molecular variance (Fig. 3D).

The pairwise *F*_*ST*_ values ranged from 0.14 to 0.15 in the three major clusters and from 0.17 (between sub-clusters I and II) to 0.47 (between sub-clusters III and VI) in the eleven sub-clusters (Table 1). AMOVA showed significant (*P* < 0.01) genetic variation among and within the major as well as the sub-clusters (Supplementary Table S4).

**Table 1:** The Nei’s genetic distance (above the diagonal) and estimated fixation index (*F*_*ST*_) (below the diagonal) between the eleven sub-clusters.

SC	SC-I	SC-II	SC-III	SC-IV	SC-V	SC-VI	SC-VII	SC-VIII	SC-IX	SC-X	SC-XI
SC-I	**0.00**	0.09	0.09	0.10	0.16	0.22	0.15	0.14	0.20	0.15	0.16
SC-II	0.17	**0.00**	0.10	0.10	0.12	0.19	0.12	0.11	0.17	0.13	0.13
SC-III	0.20	0.18	**0.00**	0.12	0.17	0.23	0.17	0.15	0.21	0.17	0.17
SC-IV	0.21	0.19	0.25	**0.00**	0.11	0.17	0.11	0.12	0.16	0.08	0.13
SC-V	0.33	0.25	0.36	0.26	**0.00**	0.11	0.13	0.14	0.17	0.16	0.17
SC-VI	0.45	0.35	0.47	0.37	0.33	**0.00**	0.11	0.16	0.12	0.15	0.18
SC-VII	0.28	0.22	0.30	0.22	0.27	0.25	**0.00**	0.09	0.10	0.08	0.11
SC-VIII	0.26	0.20	0.29	0.23	0.29	0.34	0.20	**0.00**	0.15	0.12	0.14
SC-IX	0.42	0.32	0.44	0.35	0.40	0.37	0.23	0.33	**0.00**	0.15	0.18
SC-X	0.30	0.25	0.33	0.20	0.33	0.34	0.19	0.26	0.33	**0.00**	0.10
SC-XI	0.30	0.23	0.32	0.25	0.34	0.40	0.22	0.26	0.38	0.23	**0.00**

Significant values are in bold.

*SC* sub-cluster.

### Genetic diversity between and within the collections and progeny plants

**Figure 4:**
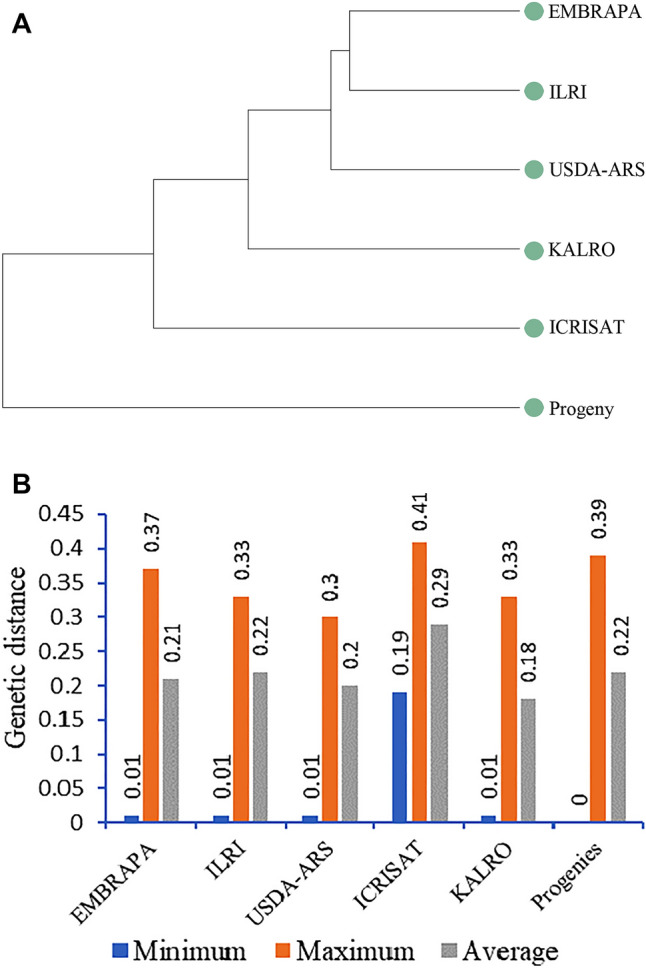
UPGMA dendrogram (**A**) depicting the genetic relationships between the five Napier grass collections and progeny plants. In (**B**) the minimum, maximum, and average Nei’s genetic distance within each of the collections and progeny plants.

The genetic diversity between and within the five collections (ILRI, EMBRAPA, USDA-ARS, ICRISAT, KALRO) and progeny plants were compared using Nei’s genetic distance^33^ and fixation index (*F*_*ST*_)^34^. The pairwise Nei’s genetic distance ranged from 0.018, between ILRI and KALRO/EMBRAPA, to 0.05, between the KALRO collection and progeny plants. Similarly, the estimated *F*_*ST*_ values ranged from 0.029, between ILRI and ICRISAT collections, to 0.101, between the KALRO collection and progeny plants (Table 3) which reflects moderate genetic variation between the collections. However, the variation among the five collections was low, the moderate variation observed was mainly between the progeny plants and the five collections (Table 2; Fig. 4A), indicating that there was unique genetic makeup in the progeny plants. Variance components calculated by AMOVA were also significant (*P* < 0.001) both between and within collections, however, higher variation was detected within (90%) than between (10%) collections (Supplementary Table S5). The range and average of Nei’s genetic distance within each collection and the progeny plants is shown in Fig. 4B. Higher within genetic diversity was observed in the ICRISAT collection (0.29 average Nei’s genetic distance), followed by the ILRI collection (0.22), and the progeny plants (0.22).

**Table 2:** Nei’s genetic distance (above the diagonal) and estimated fixation index (*F*_*ST*_) (below the diagonal) between the five Napier grass collections and progeny plants.

Collection	Progeny	EMBRAPA	USDA-ARS	ICRISAT	ILRI	KALRO
Progeny	0.000	0.040	0.040	0.041	0.035	0.050
EMBRAPA	0.079	0.000	0.021	0.032	0.018	0.029
USDA-ARS	0.071	0.031	0.000	0.037	0.022	0.039
ICRISAT	0.071	0.050	0.048	0.000	0.029	0.040
ILRI	0.067	0.032	0.024	0.029	0.000	0.018
KALRO	0.101	0.058	0.072	0.072	0.034	0.000

**Figure 5:**
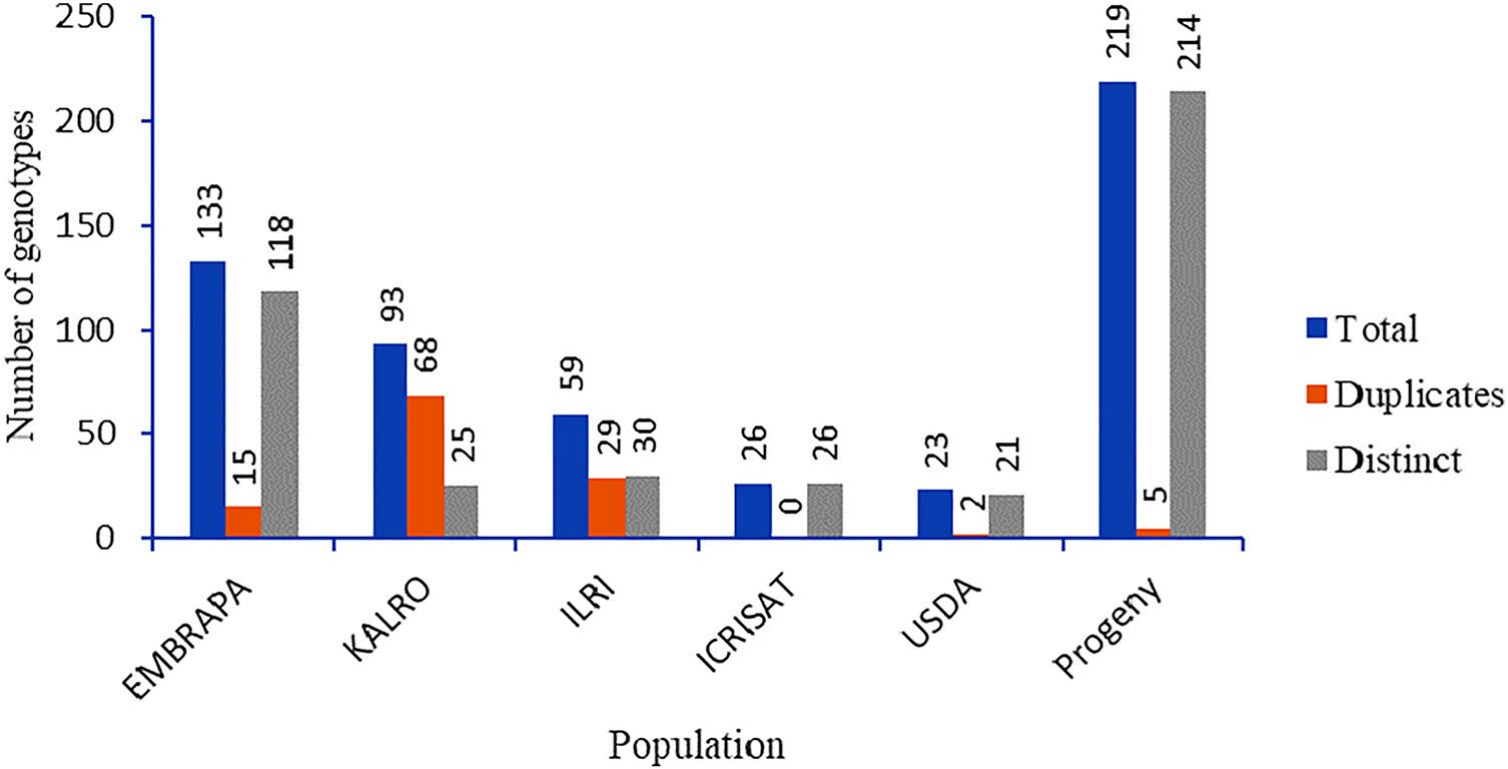
Distinct Napier grass genotypes and potential duplicates detected in each of the five collections and progeny plants.

Potential duplicate genotypes in each collection were identified using Nei’s genetic distance at a threshold of 0.025, which was equivalent to the identity-by-descent (IBD) kinship threshold (k ≥ 0.45) for self, or duplicate identification. A greater number of potential duplicates was detected in the KALRO collection, while all genotypes from ICRISAT were distinct (Fig. 5). Across collections, the largest overlap was between the ILRI and KALRO collections, followed by the KALRO and EMBRAPA, and the ILRI and EMBRAPA collections (Table 3). From the overall collection of 574 genotypes composed of the six collections, a total of 432 distinct (unique) genotypes that could be used in downstream genetic studies were selected (Supplementary Table S6).

**Table 3:** Number of duplicated genotypes between collections.

Collection	EMBRAPA	KALRO	ILRI	ICRISAT	USDA-ARS	Progeny
EMBRAPA	0					
KALRO	9	0				
ILRI	7	45	0			
ICRISAT	0	0	0	0		
USDA-ARS	3	4	3	0	0	
Progeny	0	0	0	0	0	0

### Parentage analysis in the progeny plants

We estimated the parentage of the 219 progeny plants, raised from 13 open pollinated seed-setting ILRI genotypes, using identity-by-descent (IBD)^35^ and Mendelian-inconsistent errors^36, 37^ analyses. First, we examined the genetic relationship between the recorded seed parents and progeny plants by the IBD method of the maximum likelihood (ML) relatedness estimator^35^, and these were corrected based on the IBD result whenever there was a discrepancy between the recorded seed parent and the IBD result.

**Figure 6:**
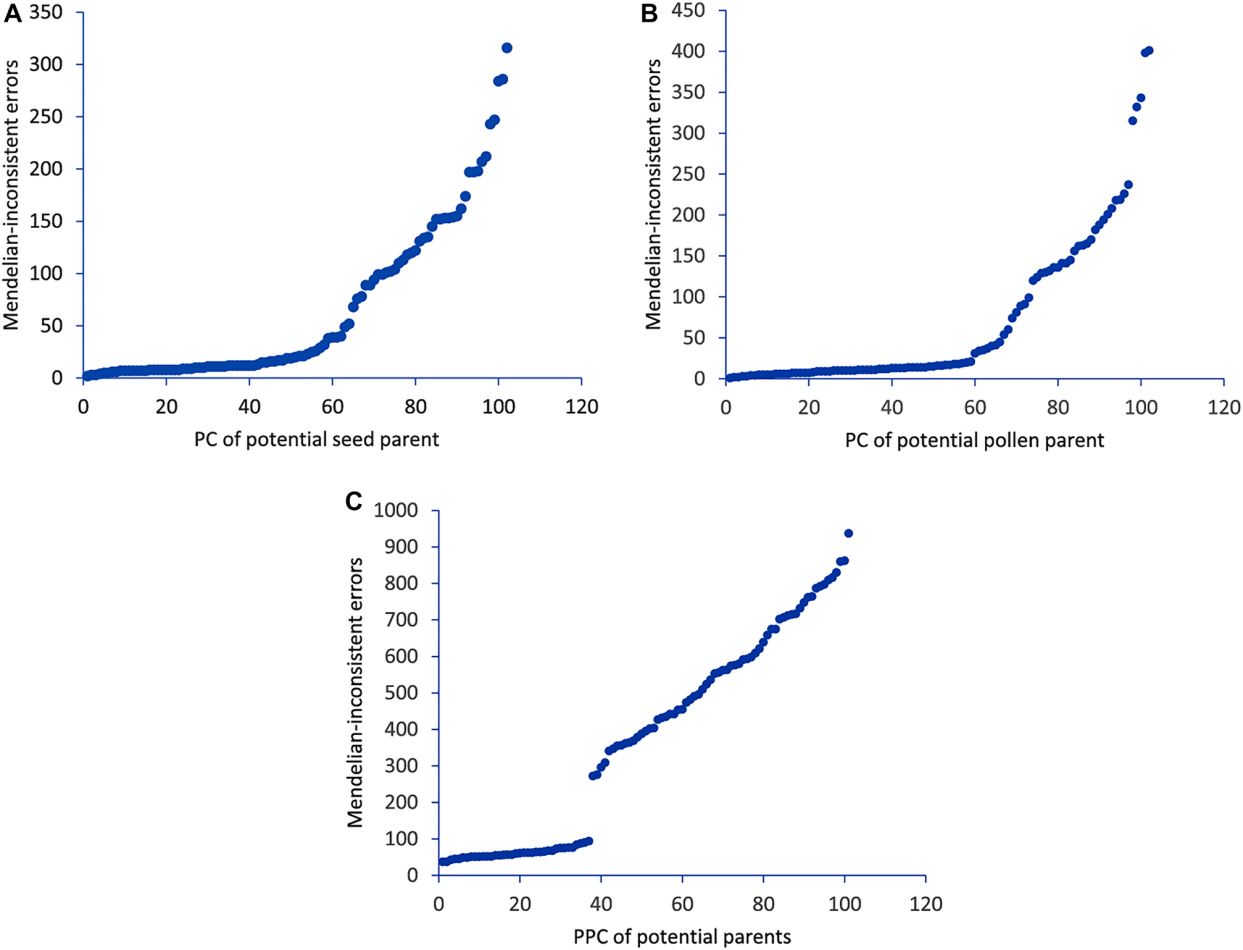
A gap in Mendelian error counts calculated on 133 progeny plants for which the recorded seed parents were confirmed by IBD analysis. (**A**) for PC1 (parent–child relationship for potential seed parents), (**B**) for PC2 (parent–child relationship for potential pollen parents), (**C**) for PPC (parent-parent–child relationship for possible crosses or as potential complete parents).

The most likely pollen donor of the progeny plants was analyzed by counting the Mendelian-inconsistent errors, considering a low percentage of errors as an indication of a putative parent–offspring relationship^36, 37^. The Mendelian error counts ranged from 2 to 316 for PC1 (parent–child relationship for potential seed parent), 1 to 401 for PC2 (parent–child relationship for potential pollen parent), and 37 to 937 for PPC (parent–parent–child relationship for possible crosses or as potential complete parents), respectively. In general, we identified or confirmed seed parents, potential pollen parents, and possible crosses as complete parents for 189 (86%) of the progeny plants (Supplementary Table S5). In addition, we classified the estimated relationships into strong-evidence vs weak-evidence based on the Mendelian error counts of the progeny plants for which the recorded seed parents were confirmed by the IBD analysis and determined a threshold for each relationship. A distinct gap in Mendelian error counts was observed at about 52 for PC1, at about 99 for PC2, and at about 94 for PPC, however, the gap for PC1 and PC2 was less clear compared to the gap for PPC (Fig. 6). Therefore, the Mendelian error counts for 53 (28%) of the PPC relationships that showed less than 94 Mendelian error counts were considered as real parents with strong evidence (Supplementary Table S7). We identified eleven ILRI genotypes as potential seed and pollen parents, however, none of the progeny plants showed parent–child relationships with two of the recorded ILRI parent genotypes (ILRI_P2_16789 and ILRI_P4_16783). ILRI_P1_1026 was the dominant pollen donor, followed by ILRI_P9_16821, ILRI_P8_16803, and ILRI_P7_16837; while ILRI_P11_16810 was the most infrequent pollen donor.

### Markers-trait associations

**Figure 7:**
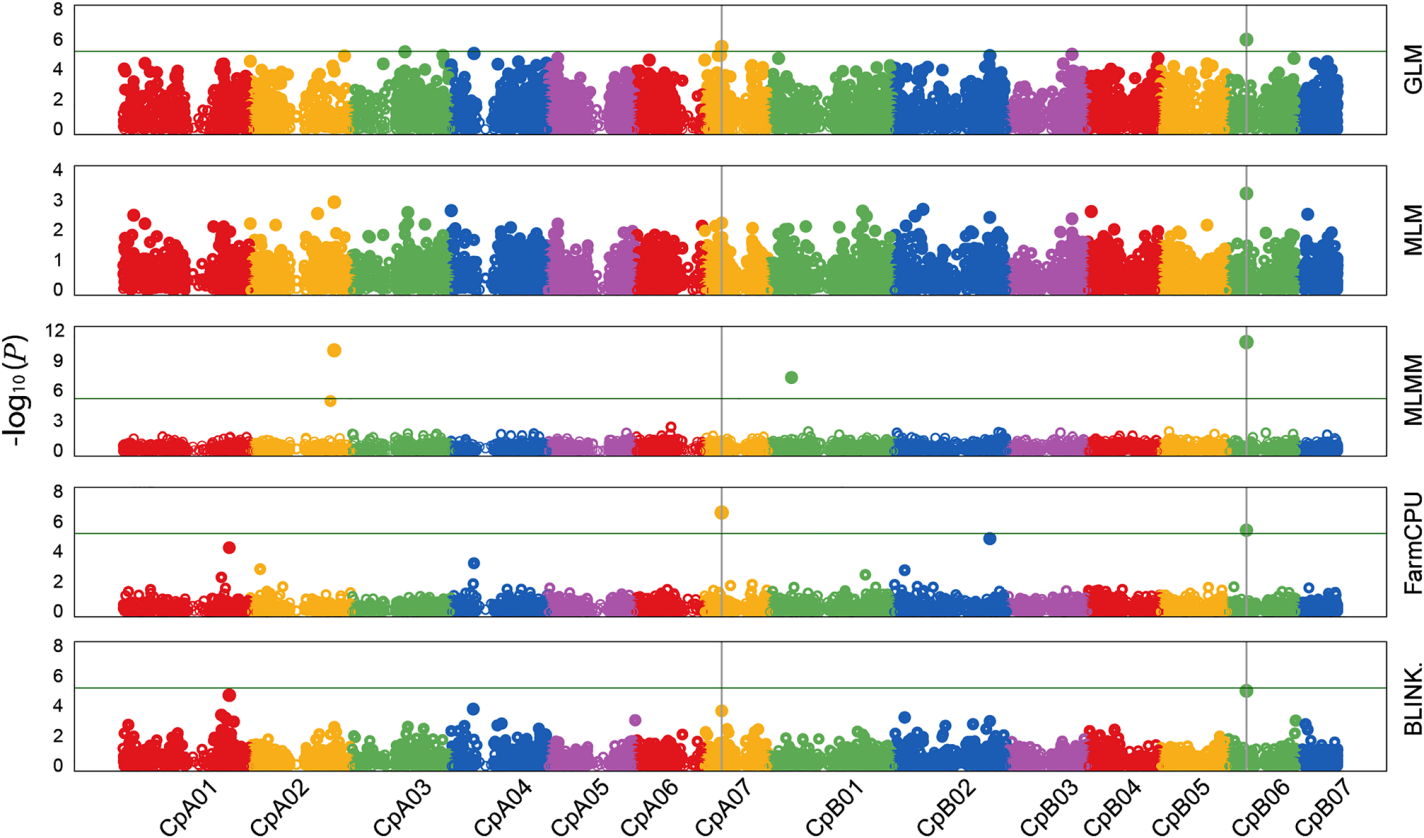
Manhattan plots showing the distribution of significant marker-trait associations identified in the Napier grass genome for forage biomass yield. The vertical axis at the left represents the − log10 *p*-value, and markers and their chromosome positions are shown on the horizontal axis. The five GAPIT models (GLM, MLM, MLMM, FarmCPU, BLINK) used are shown on the right vertical axis.

A genome wide association study (GWAS), using quality filtered DArTseq markers (12,214 SNPs and 3,869 SilicoDArTs) and field phenotype data measured on 85 Napier grass genotypes, identified seven markers (6 SNPs and 1 SilicoDArT) significantly associated with fresh biomass yield. The associated markers mapped on chromosomes CpA01, CpA02, CpA03, CpA07, CpB01, CpB02, and CpB06 and explained 0.21–21% of phenotypic variation (Table 4). Significantly, two SNP markers, on B06 and A07, were detected by at least four of the five GAPIT models employed and together accounted for more than one-third of the phenotypic variation (Table 4; Fig. 7).

**Table 4:** Markers significantly associated with forage biomass yield, their genomic positions, contrasting alleles, and minor allele frequency.

Marker	Type	Chr	Pos	Allele	MAF	*P*-value					R^2^	Effect
						GLM	MLM	MLMM	FarmCPU	BLINK		
100838531 |F| 0–8:C>A-8:C>A	SNP	CpB06	28749415	C/A	0.37	9.60E−07	5.83E−04	1.24E−11	3.51E−06	9.11E−06	21.00	2.02
100801114 |F| 0–20:C>T-20:C>T	SNP	CpA07	26898031	C/T	0.22	2.84E−06	0.0056	NA	2.31E−07	1.96E−04	15.00	− 0.914
100809503 |F| 0–9:C>T-9:C>T	SNP	CpA03	82975648	C/T	0.33	6.29E−06	NA	NA	NA	NA	0.21	− 1.048
100472665	SilicoDArT	CpA02	131154784	0/1	0.31	NA	NA	8.28E−11	NA	NA	16.00	0.71
100760175 |F| 0–60:G>A-60:G>A	SNP	CpB01	36427708	G/A	0.26	NA	NA	4.40E−08	NA	NA	7.70	0.78
100680666 |F| 0–26:C>A-26:C>A	SNP	CpB02	17060145	C/A	0.23	NA	NA	NA	4.06E−06	NA	5.90	0.857
100815322 |F| 0–24:G>T-24:G>T	SNP	CpA01	153751512	G/T	0.23	NA	NA	NA	NA	2.63E−06	2.71	− 0.154

*Chr* chromosome, *Pos* position in a chromosome, *MAF* minor allele frequency.

## Discussion

It is thanks to the diversity of plant genetic resources serving as a source of new alleles which support the development of new cultivars with desirable qualities, including breeder’ and farmers’ preferred traits such as high biomass yield, high nutritional quality, larger seeds, and pest and disease resistance^1, 4, 31^. In order to assess the level of global genetic variation in Napier grass, a diverse set of genotypes acquired from five international institution collections was used in this study. Each collection had a different number of genotypes, ranging from 23 in USDA-ARS to 133 in EMBRAPA. In addition, progeny plants raised from 13 open pollinated seed-setting ILRI genotypes were evaluated for genetic diversity and compared to the diversity held in the collections. These were included with the aim of enhancing the genetic diversity and generating information useful for designing breeding strategies for Napier grass. Each collection represented a different region of the world; for example, the ILRI and KALRO collections were mostly from Africa^18, 38^, while the EMBRAPA materials were mainly from Brazil^39, 40^. Similarly, most of the genotypes from ICRISAT’s collection had an Asian origin^41^. The USDA-ARS materials were from Latin-America (mainly from Puerto Rico), Africa (mainly from Kenya), and Tifton (genotypes derived from the cultivar “Merkeron” in a breeding program)^28, 42^.

We employed the GBS method of the DArTseq technology^43^, an efficient and cost-effective sequencing technology that concurrently generates dominant SilicoDArT and co-dominant SNP markers (https://www.diversityarrays.com/services/dartseq/) with a high density and genome coverage. Similar to our previous reports^5, 18, 44^, we obtained a large number of markers, 114,881 SNPs and 46,293 SilicoDArTs, distributed over the fourteen chromosomes of the Napier grass genome. More than 80% of the markers (from both marker types) were able to be mapped onto the genome, approximately equally onto the A' and B sub genomes^19^. We applied strong SNP filtering criteria by setting a maximum of 10% missing values, a minimum of 5% minor allele frequency, a maximum of 0.5 linkage-disequilibrium (LD) value to ensure independent markers and, markers that were able to be mapped across the genome. The filtered polymorphic markers, with average PIC and He values of 0.26 and 0.32, respectively, were used in the genetic diversity analysis.

### Genetic diversity in worldwide Napier grass collections

The Napier grass genotypes from the worldwide collections and progeny plants examined in this study demonstrated substantial genetic variation with an average value of 0.23 (0 to 0.41 range) Nei’s genetic diversity coefficients^33^. These genetic distance values were greater than the range that Wanjala et al.^45^ reported for 281 Napier grass genotypes. The high genetic variability detected could be attributed to the high levels of heterozygosity that exists in Napier grass due to its self-incompatibility and obligate outcrossing nature^26^. Napier grass is a highly heterozygous tetraploid species with a broad parental diversity and rich gene pool^23, 40^.

Furthermore, we detected three major clusters and eleven sub-clusters that were significantly different from each other, based on the admixture model in STRUCTURE, hierarchical clustering, PCA, and AMOVA analyses. The *F*_*ST*_ values ranged from 0.14 to 0.15 between the three clusters and 0.17 to 0.47 between the 11 sub-clusters. This substantial genetic variation was detected both within and between clusters, while within-cluster variation was slightly larger (54%) than between-cluster variation (46%). Generally, outcrossing species show higher levels of genetic variation among genotypes and less population divergence, compared to self-pollinating species^46^. Similar results were reported in other outcrossing species such as Rhodes grass (*Chloris gayana*)^47^ and perennial ryegrass (*Lolium perenne*)^48^. Nevertheless, about 46% of the genetic variation was detected between the eleven sub-clusters. This higher divergence could be explained by the clonal propagation of Napier grass through stem cuttings, which reduces genetic drift and gene flow among genotypes within a population^45, 46, 49^. Genotype adaptation to specific environmental conditions might have also contributed to the high genetic diversity among the clusters, given that the genotypes were collected from different parts of the world.

With an *F*_*ST*_ value of about 0.47, cluster III (which consisted of progeny plants, mainly from NS-11) and cluster VI (mainly from the ILRI and KALRO collections) had the highest genetic distance, followed by between cluster I (progeny plants, mainly from two seed parents, NS-6 and NS-9) and cluster VI, and cluster III and IX (comprised mainly of genotypes from EMBRAPA, ILRI, and KALRO). The results indicated a divergence between the collections and progenies, showing the potential of the progeny plants, that were produced from open pollination, for enhancement of genetic diversity in Napier grass collections and potential for future use in breeding programs, such as heterosis breeding. Heterotic contributions to increased biomass production in the F1 hybrids of Napier grass have previously been reported^50, 51^.

The ILRI genotypes were distributed across sub-clusters, mainly in sub-clusters II, VI, VII, IX, and the admixture groups, and their clustering was mostly consistent with our previous report^18^. The genotypes in cluster II are low to medium biomass yielders, whereas the genotypes in clusters VI and VII are medium to high biomass yielders^5^. From the EMBRAPA collection, the majority of the elite lines (indicated by the acronym ‘CNPGL’ in Supplemental Table S3) and the purple genotypes were in the admixture-2 group, while most of the genebank materials (indicated by the acronym ‘BAGCE’) were in Cluster VIII, which was mostly consistent with our previous report^18^ and reflected the breeding history of the collection. BAGCE_30 from cluster VIII and CNPGL_92-66-3 from the admixture group were reported to be high biomass yielding and drought tolerant genotypes under Ethiopian conditions^5^. Cluster V contained genotypes mainly from the KALRO collection, including Kakamega 1 and 2 genotypes, which have been reported to be tolerant to Napier grass head smut disease^52^. This cluster included two important ILRI genotypes (ILRI_16791 and ILRI_16802), which have been identified for their superior biomass yields, drought tolerance, and comparatively higher protein content^5^.

Cluster XI was primarily comprised of USDA-ARS genotypes, with a few EMBRAPA genotypes. In line with the report by Harris et al.^28^, genotypes derived from the cultivar ‘Merkeron’ were clustered together in this group. ‘Merkeron’ is a well-known cultivar in the USA, released by a breeding program in Tifton, Georgia^50^ and was derived from an intraspecific cross between a high yielding clone and a dwarf leafy clone, with improved yield and disease resistance^29^. Among these, the well-known variety “Mott” (Tift N75), a dwarf leafy type selected from progeny of selfed Merkeron^30^, is included in this cluster. The variety "Mott" was introduced to ILRI, has been preserved in the genebank with accession number ILRI_15743 and distributed to farmers and national research institutes in Ethiopia. However, none of the samples collected from farmers’ fields and national research institutes, as well as the genotype labeled as ILRI_15743, clustered in this group. Similar findings were reported by Harris et al.^28^, who found that none of the "Merkeron" samples taken from the various countries where "Merkeron" was distributed were genetically identical, which possibly happened due to genetic mixing up through outcrossing.

Cluster VII consisted of most *C. purpureus* × *C. americanus* hybrids including the commercial varieties ‘Maralfalfa’^53^, ‘Super-Napier’, and the hybrids from the ILRI collection ILRI_16840, ILRI_16837, ILRI_15357^18^. ILRI_15357 showed very high genetic similarity to ‘Maralfalfa’, with more than 0.47 IBD value. Maralfalfa has been reported for its adaptability to higher altitudes (3000 m asl) and for high biomass yield (60 tn/ha) with higher protein content^53^. Many genotypes from Kiambu under the KALRO collection were also found in this cluster, perhaps suggesting that there are also hybrids held in this collection.

The progeny plants were almost exclusively distributed in clusters I, III, IV, and X, which were significantly different from each other with a range of *F*_*ST*_ values of 0.20 to 0.33 and an average value of 0.25. The progenies in cluster I and III were particularly distinct from the other clusters with an average *F*_*ST*_ value of 0.32, showing their divergence from genotypes and other progenies. Napier grass's high heterozygosity within genotypes, self-incompatibility, and obligate outcrossing nature^54^, which can increase the likelihood of developing new genetic make-up through recombination, may be responsible for this high level of diversity and population stratification in progeny plants.

### The collections displayed less genetic divergence and different levels of genotype redundancy

The collections we studied generally exhibited low to moderate genetic differentiation with an *F*_*ST*_ range of 0.024 between the ILRI and USDA-ARS collections to 0.072 between the KALRO and ICRISAT collections. The genetic variation between collections explained only about 10% of the variation as compared to 90% of the variation explained by genotypes within collections, indicating a degree of overlap and redundancy that may have resulted from sharing and exchanging materials among the genebanks. Although Napier grass genotypes are primarily maintained via vegetative multiplication using cuttings, there is still some degree of hybridization existing in nature and also controlled crossings in some collections^1, 26^. Such redundancy of genotypes between genebanks could also have arisen because each genebank or breeding program gives its own different accession numbers, as there is no standardized labelling and registration procedure. Facilitated by the International Treaty on Plant Genetic Resources for Food and Agriculture, the ILRI forage genebank has assigned a digital object identifier (DOI) to each genotype held in its genebank^18, 47^, which could be a promising solution to this issue but should then be adopted by more national and international genebanks and research institutions.

The presence of genotype overlaps and redundancy among the collections was further confirmed by Nei’s genetic distance and identity-by-descent (IBD) analysis; the largest overlap was between the ILRI and KALRO collections, followed by between the KALRO and EMBRAPA, and the ILRI and EMBRAPA collections. The substantial overlap between the ILRI and KALRO collections could be due to the close proximity of the two institutes and historical sharing of genotypes, as indicated in the genotypes name, a large number of KALRO's genotypes were sourced from the ILRI collection. This suggests that the introduction and incorporation of new genotypes to a collection should be carried out strategically to minimize duplications and capture only new genetic diversity that is distinct from the existing one, as conservation is a costly business^32^.

Within collection genotype redundancy analysis, also showed a greater number of potential duplicates in the KALRO collection whereas all genotypes from ICRISAT were distinct. While genotype redundancies and overlaps between collections from various institutions, especially those from different continents like Africa, South America, Asia, and North America, can be taken into consideration and should be encouraged to preserve genotypes with specific adaptations to a wide range of farming systems and agroecological conditions, overlap within a collection should be reduced as it imposes a conservation cost. However, it is crucial to carefully remove overlaps using molecular marker analysis supported by field phenotype and passport data whenever possible in order to preserve unique alleles in closely related genotypes.

### Greater genetic variation was detected in progeny plants

When compared to the variation among the five collections, the progeny plants were genetically separated, with *F*_ST_ values ranging from 0.067 between the progenies and ILRI collection to 0.101 between the progenies and KALRO collection. This finding further supports the potential of progeny plants to increase genetic diversity with the potential to be used in breeding programs in the future. Open-pollinated progeny selection has been one of the breeding approaches in Napier grass and has resulted in the generation of improved cultivars^1^. Progeny plants from an open-pollinated population of cultivars ‘A146’, ‘A148’ and ‘A149’ were developed in Taiwan, and the progenies produced 20% more dry matter than the seed parents, had very few hairs on the leaves, flowered late and grew more erect^1, 55^. However, the use of open pollination breeding schemes allows the control of only one-half of the genome of the resulting progeny material because contributions from pollen parents are not controlled, and the transfer of undesirable traits from unimproved lines can limit or slow the successful release of new cultivars. Therefore, Napier grass breeding programs should focus on targeted crossings that have the potential to yield higher rates of success^50^ in rapidly generating desirable genotypes and eventually culminating in hybrid breeding wherein a superior individual can be maintained by vegetative propagation after being identified.

In this study, we were able to identify potential seed and pollen parents for more than 80% of the progeny plants using identity-by-descent (IBD) analysis based on the maximum likelihood (ML) relatedness estimator and Mendelian-inconsistent errors^36, 37^. The progenies in clusters I and III were produced primarily from the ILRI_P9_16821 and ILRI_P6_16835 genomes, while the progenies in clusters IV and X were dominantly from ILRI_P7_16837 and ILRI_P13_16790 and ILRI_P1_1026 and ILRI_P8_16803 genomes, respectively. These results could offer a stronger basis for selecting superior progeny plants for further selection and improvement through heterosis breeding in Napier grass.

### Genomic regions governing forage biomass yield

A genome-wide association study identified seven QTL regions associated with forage biomass yield, which represents the above-ground fresh biomass production. The QTLs on chromosome CpB06 and CpA07 showed a strong association and were detected by five different statistical models, suggesting that they can potentially be exploited in marker-assisted selection to develop high biomass producing Napier grass varieties. A marker on chromosome CpB06 that was strongly associated with fresh and dry biomass yields and water use efficiency has previously been reported^44^.

## Conclusions and recommendations

1. This study represents the first comprehensive genetic diversity study in global Napier grass collections and revealed the presence of extensive genetic variation among Napier grass genotypes. The analysis detected major clusters and sub-clusters that were significantly different from each other with higher within cluster/sub-cluster genetic variation. The study provides a global picture of the available genetic diversity of this important tropical perennial crop and the findings could be used to guide targeted use and conservation of the diversity by breeders and genebanks. Additionally, the information can be used to determine heterotic groups for future Napier grass breeding and improvement through heterosis breeding. Given that the genotypes were collected from different parts of the world, which shows their potential adaptation to a wide range of environmental conditions, studies on genotype by environment interactions are necessary in the future.
2. The genetic variation amongst the different genebank collections studied here was generally low and various levels of genotype redundancy were detected both within and across collections. Genotype overlaps between genebanks, particularly those from different continents, should be encouraged in order to preserve genotypes with specific adaptations to a wide range of agroecological conditions. To reduce the cost of conservation, overlaps within a genebank should be minimized. However, overlaps within a genebank must be carefully removed to maintain unique alleles in closely related genotypes.
3. Higher genetic variation and divergence was detected in the progeny plants produced from open pollination, which creates new genetic makeup and diversity. Incorporating some of the distinctive progeny plants into the genebank collections will be advantageous to enhance the genetic diversity and population size of the collections. However, a few genotypes predominated as a pollen source, as suggested by the parentage analysis, which invites further research on the mechanisms of pollination in Napier grass genotypes maintained in the ILRI forage genebank.

## Methods

### Plant materials

A total of 581 Napier grass genotypes, comprising 219 progeny plants that were raised from open pollinated seeds and 341 genotypes from worldwide collections maintained by five institutes, were included in the study. These include 60 genotypes from the ILRI genebank collection, 133 genotypes from the EMBRAPA collection, 93 genotypes from KALRO, 23 genotypes from the USDA-ARS and, 32 genotypes from the ICRISAT genebank. Two commercial varieties, Maralfalfa^53^ and Super-Napier (Pakchong 1)—both reported as hybrids of Napier grass and pearl millet with high biomass yield and protein content, were included. An additional 19 genotypes, collected from different places, with the name ‘Mott” or with unknown names were also included (Supplementary Table S1).

About 40 to 50 Napier grass seeds, collected from 13 seed-bearing ILRI genotypes and maintained in the ILRI forage genebank, were obtained from the genebank and pre-germinated on agar medium containing potassium nitrate. The germinated seeds were transplanted into pots filled with soil and maintained in a screen house until they produced three to four leaves, at which point plant samples for DNA extraction were taken. On average, about 17 progeny plants from each of the 13 genotypes were sampled to assess the level of genetic diversity within and between the groups of progenies, as well as to assess the genetic diversity in progeny plants compared to the collections^27^.

The ILRI forage genebank, EMBRAPA, KALRO, ICRISAT, or USDA-ARS provided all Napier grass samples, including seeds, leaves, or DNA samples. The studies were carried out in accordance with the guidelines and legislation outlined in the IUCN Policy Statement on Research Involving Species at Risk of Extinction and the Convention on the Trade in Endangered Species of Wild Fauna and Flora. Napier grass is a popular forage species which is cultivated across the tropics and not at risk of extinction or a wild species.

### DNA extraction and genotyping

Young leaf samples from each individual plant were collected into 2 ml Eppendorf tubes, stored in an icebox containing frozen ice, and transferred to a − 80 freezer as quickly as possible. The samples were freeze-dried for about 48 h and ground into fine powder using a tissue lyser. Genomic DNA was extracted from the leaf powder using a DNeasy® Plant Mini Kit (250) (Qiagen Inc.,Valencia, CA) following the manufacturer’s procedures. The concentration and quality of the extracted DNA was assessed using a Nano-drop spectrometer (DeNovix DS-11 FX spectrophotometer) and 0.8% agarose gel electrophoresis. The DNA concentration was adjusted to 50 to 100 ng/μl in 96 well semi-skirted plates and sent to SEQART-AFRICA (https://www.seqart.net/), Nairobi, Kenya for genotyping.

The genotyping was conducted using the GBS (Genotyping By Sequencing) method of the DArTseq platform^43^, which provides combined marker identification and genotyping for SNP and SilicoDArT (presence/absence of DNA fragments in genomic representations) markers, combining the DArT complexity reduction with sequencing on the Next Generation Sequencing (NGS) platforms^18, 43^. The markers were aligned with the Napier grass genome^19^ using the short sequence fragments associated with each marker to generate information on map position of the sequences and markers across the genome. A heatmap showing the density and distribution of markers across the fourteen Napier grass chromosomes was generated using a free online tool at https://www.bioinformatics.com.cn/en. For genetic diversity analysis, polymorphic markers were filtered based on the marker’s minor allele frequency (MAF), missing values, and independence from each other (based on pairwise linkage disequilibrium, LD) using the dartR^56^ and SNPRelate^57^ R-packages in R statistical software (R Core Team, 2022).

### Genetic diversity and population structure analysis

Population structure and sub clustering among the Napier grass genotypes was assessed based on the Bayesian algorithm implemented in STRUCTURE (ver. 2.3.4) software^58^ using the filtered polymorphic markers. The burn-in time and number of iterations were both set to 100,000 with 10 repetitions, assuming hypothetical subpopulations (K) ranging from 1 to 20 in an admixture model with correlated allele frequencies. The results of the run were uploaded to the software “Structure Harvester” (http://taylor0.biology.ucla.edu/structureHarvester/) and the most likely number of subpopulations was determined by the Evanno method^59^. Genotypes with less than 60% membership probability were considered admixed. Population structure was further examined using hierarchical clustering and principal component analysis (PCA) using Poppr^60^ and adegenet^61^ R-packages. Genetic diversity was estimated using pairwise Nei’s^33^ genetic distance.

Pairwise estimates of population differentiation and divergence between clusters, sub-clusters, and collections were analysed by Analysis of MOlecular VAriance (AMOVA)^62^ and fixation index (*F*_*ST*_)^34^ in the R-packages Poppr^60^, adegenet^61^, and APE^63^. AMOVA was used for partitioning the total variance in allele frequencies within and among clusters, sub-clusters, and collections and to assess the significance level of the variations. *F*_*ST*_, which ranges from 0 to 1, assesses the degree of genetic differentiation between clusters, sub-clusters, and collections. A value of 0 indicates no differentiation while a value of 1 indicates complete differentiation. In general, an *F*_*ST*_ value of 0:00 to 0:0.05 indicates low differentiation, 0:05 to 0:0.15 indicates moderate differentiation, 0.15 to 0.25 high differentiation, while *F*_*ST*_ > 0.25 are considered to represent very high levels of differentiation^64^.

### Parentage analysis

Parentage and genetic relationship analyses were conducted using identity-by-descent (IBD) analysis based on the maximum likelihood (ML) relatedness estimator^35^ using the R package SNPRelate^57^ and by counting the Mendelian-inconsistent errors using a custom R script reported in Vanderzande et al.^36^. The IBD analysis was mainly used to confirm the genetic relationship between the recorded seed parents and progeny plants, whereas Mendelian-inconsistent errors count was used to determine any putative set of parent–child (PC) and parent-parent–child (PPC) relationships. In the Mendelian-inconsistent errors count, a low percentage of errors was used as an indication of putative parent–offspring relationships while higher percentages were indicative of more distant relationships^36, 65^.

### Field phenotyping of Napier grass genotypes

A subset of 85 Napier grass genotypes were planted in the main rain season (mid-June to mid-September 2018) using an augmented p-rep design with two replications at Addis Ababa, Ethiopia. Six stem cuttings per genotype were planted in a single row with a distance of 750 mm. After establishment, the plants were clean cut every 12 weeks of regrowth to a standard height of 50 mm above ground. Total fresh biomass yield per plant (TFW) was taken by weighing and calculating the average from three randomly selected plants per row. The average value per genotype was calculated based on 24 plants, taken from three plants per row, in two replications, across four harvests from the 2018 to 2021 cropping seasons.

### Marker trait association analysis

Marker-trait association analysis was conducted using five statistical models implemented in Genomic Association and Prediction Integrated Tool version 3 (GAPIT3)^66^. The models used include General Linear Model (GLM), Mixed Linear Model (MLM), Multiple Loci Mixed Model (MLMM), Fixed and random model Circulating Probability Unification (FarmCPU) and Bayesian information and Linkage-disequilibrium Iteratively Nested Keyway (BLINK). A total of 16,083 markers (12,214 SNPs and 3,869 silicoDArTs) were retained after filtering. The markers were filtered based on call rate (≥ 0.90), minor allele frequency (MAF ≥ 5%), missing values (NA < 10%), and their distribution across the genome.

### Supplementary Information

Supplementary Tables.

## Data Availability

Most of the datasets generated for the current study are found in the supplemental information, high density genome-wide SNP and SilicoDArT marker data on 574 Napier grass genotypes can be found at https://hdl.handle.net/10568/131571, and further data can be obtained from the corresponding author upon reasonable request. The ILRI accessions are freely available to researchers who accept the terms and conditions of the Standard Material Transfer Agreement (SMTA) of the International Treaty on Plant Genetic Resources for Food and Agriculture.
